# Association between hypertension prevalence and neighborhood characteristics in Japan using combined claims data and geographic information systems: a neighborhood correlation study from LIFE Study

**DOI:** 10.1265/ehpm.26-00095

**Published:** 2026-08-08

**Authors:** Kan Oishi, Rei Ono, Kojiro Ishii, Futoshi Oda, Megumi Maeda, Takaaki Mori, Tomoki Nakaya, Haruhisa Fukuda

**Affiliations:** 1 Faculty of Education, Saga University, 1 Honjo-machi, Saga-shi, Saga 840-8502, Japan; 2 Health and Human Performance Research Center, Doshisha University, 1-3 Tatara Miyakodani, Kyōtanabe-shi, Kyoto 610-0394, Japan; 3 National Institutes of Biomedical Innovation, Health, and Nutrition, Japan Kento Innovation Park NK Building, 3-17 Senrioka Shinmachi, Settsu-shi, Osaka 566-0002, Japan; 4 National Center for Geriatrics and Gerontology, Department of Social Science, 7-430 Morioka-cho, Obu, Aichi 474-8511, Japan; 5 Graduate School of Medicine, Kobe University, 10-2 Tomogaoka 7-Chome, Suma-ku, Kobe 654-0142, Japan; 6 Faculty of Health and Sports Science, Doshisha University, 1-3 Tatara Miyakodani, Kyotanabe-shi, Kyoto 610-0394, Japan; 7 Department of Health Care Administration and Management, Graduate School of Medical Sciences, Kyushu University, 3-1-1 Maidashi, Higashi-ku, Fukuoka 812-8582, Japan; 8 Japan Society for the Promotion of Sciences, Kojimachi Business Center Building, 5-3-1 Kojimachi, Chiyoda-ku, Tokyo 102-0083, Japan; 9 Graduate School of Environmental Studies, Tohoku University, 468-1 Aramaki-Aoba, Aoba-ku, Sendai 980-8572, Japan; 10 Department of Earth Science, Graduate School of Science, Tohoku University, 6-3 Aramaki-Aoba, Aoba-ku, Sendai 980-8578, Japan; 11 Center for Cohort Studies, Graduate School of Medical Sciences, Kyushu University, 3-1-1 Maidashi, Higashi-ku, Fukuoka 812-8582, Japan

**Keywords:** Socioeconomic status, Educational attainment, Green space, Neighborhood environment, Japan

## Abstract

**Background:**

Hypertension is prevalent and is influenced by modifiable factors. Increasing attention has focused on community-based health promotion. However, studies on the relationship between neighborhood characteristics and hypertension prevalence are limited. This study combined medical claims and geographic information system data to identify neighborhood characteristics associated with hypertension.

**Methods:**

The study population included participants enrolled in the National Health Insurance or Medical Care System for the Elderly in both 2017 and 2021, and their 2021 data were aggregated at the elementary school district level. Hypertension was defined using the International Statistical Classification of Diseases and Related Health Problems-10 codes I10–I15. Neighborhoods were defined by elementary school districts. For each school district, hypertension prevalence, the proportions of participants aged <65 years, 65–74 years, and ≥75 years, and the proportion of men were calculated. Neighborhood characteristics included college graduation rate, area deprivation index, annual average temperature, annual total precipitation, average slope angle, number of destination types, and densities of public places. Quasi-Poisson regression analyses were conducted with the number of hypertension cases as the dependent variable, neighborhood characteristics as explanatory variables, the proportions of participants aged 65–74 years and ≥75 years, and the proportion of men (in the overall analysis) as covariates, and the number of participants as an offset term. For the quasi-Poisson regression analyses, a Bonferroni-adjusted p-value < 0.05 was considered statistically significant.

**Results:**

The study covered 424 elementary school districts across 14 municipalities in five prefectures in western Japan. The mean number of participants per district was 2,248 (men: 946, women: 1,302), with an average age of 67 years (men: 66; women: 68). The mean hypertension prevalence was 51% (men: 53%; women: 50%). In the combined and female-only analyses, the neighborhood proportion of university graduates and green space coverage were significantly negatively associated with hypertension prevalence. In the male-only analysis, the proportion of university graduates, green space coverage, and park density showed significant negative associations.

**Conclusions:**

Neighborhood socioeconomic and physical environmental characteristics were associated with hypertension prevalence in Japan, particularly educational attainment and green space coverage, suggesting that social and physical environmental attributes may contribute to community-level cardiovascular health.

## Background

Globally, approximately one in three adults aged 30 to 70 years are estimated to have hypertension, and it is a major risk factor for mortality worldwide [1, 2]. In Japan, the number of individuals with hypertension is estimated at around 43 million, making it the most prevalent lifestyle-related disease [3]. A pooled analysis of 13 Japanese cohort studies showed that high blood pressure is associated with an increased risk of mortality across all sexes and age groups [4]. Moreover, Ikeda et al. [5] reported that hypertension is the second leading risk factor for mortality in Japan.

Against this backdrop, the prevention and management of hypertension represent urgent public health challenges domestically and globally.

At the individual level, well-established risk factors for hypertension include excessive sodium intake, insufficient physical activity, and obesity [1, 2]. In recent years, however, increasing attention has been paid to neighborhood characteristics that may directly or indirectly influence health through individual behaviors [6–8]. Similarly, systematic reviews have shown that neighborhood safety and poverty are consistently associated with hypertension [9, 10]. However, despite growing evidence linking neighborhood characteristics to hypertension, several important limitations remain. First, few studies have comprehensively and simultaneously examined multiple objectively measured neighborhood characteristics within a unified analytical framework. Neighborhood characteristics are interrelated; therefore, objectively and simultaneously evaluating multiple characteristics within a unified analytical framework helps identify those independently associated with hypertension prevalence. Second, no studies have directly examined the association between neighborhood characteristics and hypertension in Japan. Compared with Western countries, where this type of research is well established, Japan differs substantially in its built environment, population density, urban structure, and social context [11, 12]. Furthermore, the average salt intake among Japanese adults is approximately 9.4 g/day [13], far exceeding the 5 g/day recommended by the World Health Organization [1, 2], reflecting the traditionally high-salt characteristics of Japanese dietary habits. Furthermore, the prevalence of obesity in Japan, as defined by standard criteria, is substantially lower than in Western countries (approximately 20% in many Organisation for Economic Co-operation and Development countries versus <5% in Japan [14]). Individuals in East Asia may develop cardiometabolic risk at lower body mass index thresholds than those in Western countries [15]. These differences in physiological and lifestyle factors, and in urban form and architectural characteristics, raise concerns about the direct applicability of findings from Western environments to the Japanese context.

In this context, there is a clear need for a comprehensive, population-based analysis that simultaneously examines multiple objectively measured neighborhood characteristics and accounts for their interrelationships in Japan. Therefore, the present study aimed to identify neighborhood characteristics independently associated with hypertension prevalence in Japan by integrating medical claims data with geographic information system (GIS)-based indicators.

## Methods

### Study design

This study employed a cross-sectional ecological study design.

### Study data

This study utilized data from the Longevity Improvement & Fair Evidence (LIFE) Study, a database project initiated and managed by Kyushu University since 2019. The LIFE Study aims to extend healthy life expectancy and reduce health disparities in Japan [16]. It includes medical and long-term care claims data from participating municipalities to create a comprehensive database. Medical claims data contain detailed billing statements for medical services, including diagnoses and procedures performed during healthcare visits [17]. The data cover residents enrolled in either of Japan’s two public health insurance systems: the National Health Insurance (NHI) and the Medical Care System for the Elderly (MCSE). The NHI primarily covers self-employed individuals, part-time workers, retirees, and their dependents aged 0–74 years, accounting for >80% of the population aged 65–74. The MCSE covers virtually all participants aged ≥75 years. The number of municipalities participating in the LIFE Study has varied over time, depending on contractual agreements with each municipality.

### Target areas

This study included 424 elementary school districts across 14 municipalities, primarily in western Japan, all contracted under the LIFE Study. All districts in the target municipalities were included in the analysis.

### Medical claims data processing

The study included participants enrolled in either the NHI or the MCSE in both 2017 and 2021 within the target region. This restriction was applied to include participants who were likely to have resided in the target region for a certain period and thus had potential exposure to the neighborhood environment. For these participants, 2021 data were used to calculate the total number of participants, the number of participants with hypertension, the proportions of participants aged <65 years, 65–74 years, and ≥75 years, and the proportion of men for each school district. The total number of participants, the number of participants with hypertension, and the proportions of participants aged <65 years, 65–74 years, and ≥75 years were calculated for the overall study population and stratified by sex. Depending on the analysis, hypertension was analyzed as either a prevalence or a count. Hypertension was defined using the International Statistical Classification of Diseases and Related Health Problems-10 codes I10–I15.

### GIS processing

All calculations of neighborhood characteristics within the study areas were conducted using ArcGIS Pro 3.0.3 (Esri Japan, Tokyo, Japan). School districts were used as the neighborhood unit. School districts have previously provided valuable insights regarding neighborhood delineation in Japan [18–20], and we considered them an appropriate spatial scale for this analysis. Neighborhood characteristics included the proportion of university graduates, the Area Deprivation Index (ADI), monthly average temperature (normal value), monthly total precipitation (normal value), average slope angle, number of destination types, green space coverage, and park, sports facility, population, intersection, dead-end, road, station, bus stop, hospital, fast-food restaurant, convenience store, supermarket, café, other grocery store, and other food service establishment densities. These variables were selected to comprehensively capture neighborhood socioeconomic, social environment, and physical (natural and built) environment characteristics relevant to health [21].

Regarding the university graduation rate, the proportion of individuals aged ≥15 years who graduated from university or completed graduate school was derived based on polygon data (Esri Japan Co., Ltd., 2022) from the 2020 Population Census [22] for each block (cho-cho-aza). The census provides information on the total population, number of people by educational attainment, number of individuals with unknown education levels, and the population aged ≥15 years within each block. Subsequently, population data for each category at the block level were spatially overlaid with polygon data representing the school district (Geo-K LLC, Fukuoka, Japan). The population for each category within each school district was then estimated according to the proportion of overlapping area between census districts and school district polygons. This approach has been described in detail elsewhere [23]. Similarly, population density was calculated by weighting the block polygon data by the proportion of overlapping area and dividing the estimated total population by the corresponding school district area.

Additionally, the Japanese version of the ADI [24] was used as an indicator of neighborhood-level socioeconomic conditions. Previous studies employing the same ADI have demonstrated its associations with various health indicators [25–27]. The ADI was calculated at the block level using data from the 2020 Population Census. The ADI values at the cho-cho-aza level and the number of households were spatially overlaid with school district polygon data. The number of households within each school district was estimated according to the proportion of overlapping area between census blocks and school districts. Finally, a weighted average ADI was computed for each school district, using the number of households as weights. The formula for calculating the weighted average was as follows:

Where, “d” represents a school district, “i” indexes each spatial unit (e.g., cho-cho or census block) belonging to school district *d*, and “H” denotes the number of households. The detailed procedure has also been described in a previous study [23].

Monthly average temperature and monthly total precipitation normals were obtained from the National Land Numerical Information [28, 29]. Road density values were derived from 1-km mesh (third-order mesh) units provided by the same source [30].

Average slope angle values were based on 10-m mesh (fifth-order mesh) units from the National Land Numerical Information [31, 32]. These mesh-based data were then area-weighted by

$${ADI}_{d}=\sum_{i=1}^{n}(H_{i}\cdot {ADI}_{i})/\sum_{i=1}^{n}H_{i}$$

school district to calculate the mean values within each school district. For green space coverage, the Normalized Difference Vegetation Index (NDVI) was employed. The NDVI visualizes areas with active vegetation and is derived from Sentinel-2 satellite imagery developed by the European Space Agency. NDVI is originally defined on a scale from −1 to +1; in the present study, NDVI values were multiplied by 1000 and stored as integer values. Accordingly, polygons with NDVI values > 120 (equivalent to NDVI > 0.12 on the original scale) were extracted as vegetated areas, following previous studies [33, 34]. We used the dataset updated in December 2019, and the spatial resolution of the NDVI raster was 10 m × 10 m. Areas overlapping with polygons representing forest regions [35] and farmland (parcel polygons [36]) were subsequently excluded. Finally, by overlaying the NDVI-derived green space polygons onto the school district boundaries, the proportion of green space area within each school district was calculated.

Based on previous research, there are 18 destination types [37]: convenience stores (Nihon Soft Sales Co., Ltd., Tokyo, Japan), supermarkets and department stores (Nihon Soft Sales Co., Ltd., Tokyo, Japan), other grocery stores (Nihon Soft Sales Co., Ltd., Tokyo, Japan), bookstores (Nihon Soft Sales Co., Ltd., Tokyo, Japan), pharmacies (Nihon Soft Sales Co., Ltd., Tokyo, Japan), government offices [28, 29], assembly facilities [28, 29], libraries [38, 39], cafes and coffee shops (Nihon Soft Sales Co., Ltd., Tokyo, Japan), fast-food restaurants (Nihon Soft Sales Co., Ltd., Tokyo, Japan), other restaurants (Nihon Soft Sales Co., Ltd., Tokyo, Japan), gyms and fitness clubs (Nihon Soft Sales Co., Ltd., Tokyo, Japan), parks [31, 32], post offices [38, 39], banks (Nihon Soft Sales Co., Ltd., Tokyo, Japan), medical facilities (Kokusai Kogyo Co., Ltd., Tokyo, Japan), dry cleaners (Nihon Soft Sales Co., Ltd., Tokyo, Japan), and barbershops and beauty salons (Nihon Soft Sales Co., Ltd., Tokyo, Japan).

Finally, we mapped the points for the remaining 13 types of built environmental factors and calculated the number per area (density) by dividing by the area of each school district. Point data for parks, stations, and buses were obtained from the Ministry of Land, Infrastructure, Transport, and Tourism. Point data for sports facilities, fast-food restaurants, convenience stores, supermarkets, cafes, other food stores, and other restaurants were obtained from the Green Page database (Nihon Soft Sales Co., Ltd., Tokyo, Japan), which is based on telephone directories. Point data for hospitals were obtained from PAREA-Medical (Kokusai Kogyo Co., Ltd., Tokyo, Japan). Point data for intersections and dead-end streets were defined as “points with three or more branches” and “dead-end streets without branches,” respectively, by referring to ArcGIS Geo Suite Road Network (Esri Japan Co., Ltd., Tokyo, Japan).

### Statistical analysis

All analyses were conducted at the neighborhood (school district) level, overall and by gender. Descriptive statistics were presented as mean ± standard deviation. Partial correlation analyses, adjusted for the proportions of participants aged 65–74 years and ≥75 years, with participants aged <65 years as the omitted age group, and for the proportion of men in analyses combining men and women, were used as exploratory analyses to examine associations between hypertension prevalence and neighborhood characteristics. For the partial correlation analyses, unadjusted p-values are reported, and a two-sided p < 0.05 was used as the threshold for statistical significance. Quasi-Poisson regression analyses were then conducted with the number of hypertensive participants as the dependent variable, neighborhood characteristics as independent variables, and the number of participants as an offset term. The proportions of participants aged 65–74 years and ≥75 years, with participants aged <65 years as the omitted age group, and the proportion of men (for analyses combining both sexes) were included as covariates. The quasi-Poisson model was selected to account for overdispersion in the dependent variable. For the quasi-Poisson regression analyses, a two-sided p < 0.05 was considered statistically significant, with Bonferroni-adjusted p-values applied for multiple comparisons.

## Results

Table 1 presents the characteristics (mean ± standard deviation) of each variable across the study areas. The total number of participants was 2248 ± 1040, consisting of 946 ± 424 men and 1302 ± 621 women. The mean prevalence of hypertension was 53.3 ± 5.5% in men and 50.1 ± 7.0% in women. Table 2 summarizes the descriptive statistics of neighborhood characteristics for each region.

**Table 1:** Mean and SD from medical claims data for each neighborhood area.

**Variables from medical claims data (N^a^ = 424)**	**Mean**	**(SD)**
Overall		
Number of participants, n^b^	2248	(1040)
Number of cases, n^b^	1162	(562)
Prevalence, %	51.4	(6.1)
Mean age, years	67	(5)
Proportions of participants aged <65 years (%)	28.3	(8.6)
Proportions of participants aged 65–74 years (%)	29.2	(10.0)
Proportions of participants aged ≥75 years (%)	42.5	(15.1)
Proportion of men, %	57.1	(2.0)
Men		
Number of participants, n^b^	946	(424)
Number of cases, n^b^	505	(233)
Prevalence, %	53.3	(5.5)
Mean age, years	66	(5)
Proportions of participants aged <65 years (%)	30.8	(8.9)
Proportions of participants aged 65–74 years (%)	29.4	(9.7)
Proportions of participants aged ≥75 years (%)	39.8	(14.6)
Women		
Number of participants, n^b^	1302	(621)
Number of cases, n^b^	657	(332)
Prevalence, %	50.1	(7.0)
Mean age, years	68	(5)
Proportions of participants aged <65 years (%)	26.5	(8.7)
Proportions of participants aged 65–74 years (%)	29.0	(10.4)
Proportions of participants aged ≥75 years (%)	44.5	(15.8)

SD, standard deviation.

^a^number of neighborhood areas.

^b^number of participants in the neighborhood area.

**Table 2:** Mean and SD from GIS for each neighborhood area.

**Variables from GIS (N^a^ = 424)**	**Mean**	**(SD)**
ADI	6.5	(0.9)
College graduation rate, %	26.7	(10.0)
Density of parks, /km^2^	4.2	(3.6)
Density of sports facilities, /km^2^	0.9	(1.7)
Population density, persons/km^2^	6981	(6204)
Number of destination types, types/km^2^	11	(4)
Density of intersections, /km^2^	160	(109)
Density of dead-ends, /km^2^	31	(29)
Density of roads, km/km^2^	16.9	(9.9)
Density of train stations, /km^2^	0.4	(0.8)
Density of bus stops, /km^2^	6.1	(4.9)
Density of hospitals, /km^2^	0.4	(0.9)
Average slope, °	5.1	(5.0)
Green space coverage, %	25.5	(16.6)
Density of fast-food outlets, /km^2^	0.4	(1.0)
Density of convenience stores, /km^2^	2.0	(3.1)
Density of supermarkets, /km^2^	0.9	(1.4)
Density of other grocery stores, /km^2^	5.8	(8.8)
Density of other restaurants, /km^2^	11.9	(27.5)
Density of cafés, /km^2^	0.6	(1.5)
Annual average temperature, °C	15.9	(0.8)
Annual total precipitation, mm	1580	(275)

SD, standard deviation; GIS, geographic information system; ADI, areal deprivation index.

^a^number of neighborhood areas.

Table 3 shows the results of partial correlation analyses between hypertension prevalence and neighborhood characteristics, adjusted for the proportions of participants aged 65–74 years and ≥75 years, and in the combined analysis, for the proportion of men. In the combined analysis, ADI was significantly positively correlated with hypertension prevalence. In contrast, the proportion of university graduates, park and bus stop densities, and green space coverage were significantly negatively correlated with hypertension prevalence. In the male-only analysis, ADI, intersection and dead-end densities showed significant positive correlations with hypertension prevalence, whereas the proportion of university graduates, number of destination types, park and bus stop densities, and green space coverage each showed significant negative correlations. In the female-only analysis, ADI was significantly positively correlated with hypertension prevalence. In contrast, the proportion of university graduates, number of destination types, park, sports facility, bus stop, and café densities, and green space coverage were significantly negatively correlated.

**Table 3:** Partial correlation analyses examining the relationship between hypertension prevalence and each neighborhood characteristic.

**Neighborhood characteristics**	**Overall** **(N^a^ = 424)^b^**		**Men** **(N^a^ = 424)^c^**		**Women** **(N^a^ = 424)^c^**	
	***r***	***p***	***r***	***p***	***r***	***p***
ADI	**0.416**	**<0.001**	**0.360**	**<0.001**	**0.430**	**<0.001**
College graduation rate, %	**−0.497**	**<0.001**	**−0.424**	**<0.001**	**−0.572**	**<0.001**
Density of parks, /km^2^	**−0.122**	**0.012**	**−0.125**	**0.010**	**−0.193**	**<0.001**
Density of sports facilities, /km^2^	−0.028	0.561	−0.049	0.313	**−0.110**	**0.024**
Population density, persons/km^2^	−0.008	0.875	0.038	0.434	−0.095	0.051
Number of destination types, types/km^2^	−0.068	0.161	**−0.130**	**0.007**	**−0.137**	**0.005**
Density of intersections, /km^2^	0.059	0.227	**0.113**	**0.020**	−0.041	0.398
Density of dead-ends, /km^2^	0.095	0.052	**0.150**	**0.002**	0.072	0.140
Density of roads, km/km^2^	0.004	0.942	0.041	0.406	−0.116	0.017
Density of train stations, /km^2^	0.081	0.098	−0.023	0.633	−0.021	0.668
Density of bus stops, /km^2^	**−0.145**	**0.003**	**−0.096**	**0.050**	**−0.208**	**<0.001**
Density of hospitals, /km^2^	0.078	0.110	0.084	0.084	0.008	0.869
Average slope, °	0.004	0.942	−0.000	0.999	0.025	0.605
Green space coverage, %	**−0.236**	**<0.001**	**−0.176**	**<0.001**	**−0.189**	**<0.001**
Density of fast-food outlets, /km^2^	0.023	0.639	0.006	0.895	−0.036	0.465
Density of convenience stores, /km^2^	0.021	0.661	0.028	0.561	−0.057	0.239
Density of supermarkets, /km^2^	−0.002	0.971	−0.008	0.870	−0.087	0.073
Density of other grocery stores, /km^2^	0.054	0.265	−0.010	0.843	−0.080	0.101
Density of other restaurants, /km^2^	0.016	0.739	−0.029	0.549	−0.083	0.089
Density of cafés, /km^2^	−0.013	0.792	−0.088	0.070	**−0.128**	**0.009**
Annual average temperature, °C	0.048	0.329	−0.033	0.504	0.014	0.770
Annual total precipitation, mm	0.003	0.951	−0.003	0.953	0.052	0.284

ADI, areal deprivation index; Bold font denotes unadjusted *p* < 0.05.

^a^number of neighborhood areas.

^b^adjusted for the proportions of participants aged 65–74 years and ≥75 years, with participants aged <65 years as the omitted age group, and for the proportion of men.

^c^adjusted for the proportions of participants aged 65–74 years and ≥75 years, with participants aged <65 years as the omitted age group.

Table 4 presents the results of quasi-Poisson regression analyses. In all regression analyses, the variance inflation factor (VIF) values were below 10; therefore, multicollinearity was considered not to be present (Table 5). In the combined and female-only analyses, the proportion of university graduates and green space coverage showed significant negative associations with hypertension prevalence. In the male-only analysis, in addition to the proportion of university graduates and green space coverage, park density also showed a significant negative association with hypertension prevalence.

**Table 4:** Quasi-Poisson regression analyses examining the relationship between hypertension and neighborhood characteristics.

**Neighborhood environmental elements**	**Overall (N^a^ = 424)^b^**			**Men (N^a^ = 424)^c^**			**Women (N^a^ = 424)^c^**		
	**IRR**	**(95%CI)**	**adjusted *p***	**IRR**	**(95%CI)**	**adjusted *p***	**IRR**	**(95%CI)**	**adjusted *p***
ADI	1.001	(0.992–1.010)	1.000	1.005	(0.996–1.014)	1.000	0.993	(0.984–1.003)	1.000
College graduation rate (%)	0.997	(0.996–0.998)	**<0.001**	0.997	(0.997–0.998)	**<0.001**	0.996	(0.995–0.997)	**<0.001**
Density of parks (/km^2^)	0.998	(0.996–0.999)	0.144	0.997	(0.995–0.998)	**0.004**	0.999	(0.997–1.000)	1.000
Density of sports facilities (/km^2^)	0.998	(0.994–1.002)	1.000	0.999	(0.995–1.003)	1.000	0.998	(0.994–1.002)	1.000
Population density (persons/km^2^)	1.000	(1.000–1.000)	0.365	1.000	(1.000–1.000)	1.000	1.000	(1.000–1.000)	0.177
Number of destination types (types/km^2^)	1.000	(0.998–1.001)	1.000	1.000	(0.998–1.002)	1.000	1.000	(0.998–1.001)	1.000
Density of intersections (/km^2^)	1.000	(1.000–1.000)	1.000	1.000	(1.000–1.000)	1.000	1.000	(1.000–1.000)	1.000
Density of dead-ends (/km^2^)	1.000	(1.000–1.000)	1.000	1.000	(1.000–1.000)	1.000	1.000	(1.000–1.000)	1.000
Density of roads (km/km^2^)	1.000	(1.000–1.000)	1.000	1.000	(1.000–1.000)	0.211	1.000	(1.000–1.000)	1.000
Density of train stations (/km^2^)	1.002	(0.995–1.008)	1.000	0.997	(0.991–1.004)	1.000	1.002	(0.996–1.009)	1.000
Density of bus stops (/km^2^)	0.998	(0.997–1.000)	0.146	0.999	(0.998–1.000)	0.756	0.998	(0.997–1.000)	0.239
Density of hospitals (/km^2^)	0.997	(0.991–1.003)	1.000	0.998	(0.992–1.005)	1.000	0.997	(0.990–1.003)	1.000
Average slope (°)	1.000	(0.998–1.001)	1.000	1.000	(0.998–1.001)	1.000	0.999	(0.998–1.001)	1.000
Green space coverage (%)	0.999	(0.999–1.000)	**<0.001**	0.999	(0.999–1.000)	**0.010**	0.999	(0.999–1.000)	**<0.001**
Density of fast-food outlets (/km^2^)	1.003	(0.997–1.010)	1.000	1.002	(0.995–1.009)	1.000	1.005	(0.998–1.012)	1.000
Density of convenience stores (/km^2^)	0.998	(0.995–1.001)	1.000	0.999	(0.996–1.002)	1.000	0.997	(0.994–1.000)	1.000
Density of supermarkets (/km^2^)	1.004	(0.999–1.008)	1.000	1.003	(0.998–1.008)	1.000	1.004	(0.999–1.009)	1.000
Density of other grocery stores (/km^2^)	1.000	(0.999–1.001)	1.000	1.000	(0.999–1.001)	1.000	1.000	(0.999–1.001)	1.000
Density of other restaurants (/km^2^)	1.000	(1.000–1.000)	1.000	1.000	(1.000–1.000)	1.000	1.000	(1.000–1.000)	1.000
Density of cafés (/km^2^)	0.999	(0.995–1.003)	1.000	0.998	(0.994–1.003)	1.000	0.999	(0.994–1.003)	1.000
Annual average temperature (°C)	1.000	(0.999–1.001)	1.000	1.000	(0.999–1.001)	1.000	1.000	(0.999–1.001)	1.000
Annual total precipitation (mm)	1.000	(1.000–1.000)	1.000	1.000	(1.000–1.000)	1.000	1.000	(1.000–1.000)	1.000

IRR, incidence rate ratio; CI, confidence interval; ADI, areal deprivation index; Bold font denotes adjusted *p* < 0.05.

^a^number of neighborhood areas.

^b^adjusted for the proportions of participants aged 65–74 years and ≥75 years, with participants aged <65 years as the omitted age group, and for the proportion of men.

^c^adjusted for the proportions of participants aged 65–74 years and ≥75 years, with participants aged <65 years as the omitted age group.

**Table 5:** Variance inflation factors (VIFs) for variables included in the quasi-Poisson regression models.

**Neighborhood environmental elements**	**Overall** **(N^a^ = 424)^b^**	**Men** **(N^a^ = 424)^c^**	**Women** **(N^a^ = 424)^c^**
	**VIF**	**VIF**	**VIF**
ADI	4.140	3.604	3.737
College graduation rate (%)	5.885	4.709	4.554
Density of parks (/km^2^)	1.831	1.787	1.843
Density of sports facilities (/km^2^)	2.296	2.286	2.300
Population density (persons/km^2^)	5.580	5.571	5.492
Number of destination types (types/km^2^)	1.902	1.892	1.895
Density of intersections (/km^2^)	4.743	4.763	4.711
Density of dead-ends (/km^2^)	1.934	1.952	1.912
Density of roads (km/km^2^)	5.668	5.626	5.651
Density of train stations (/km^2^)	1.609	1.544	1.551
Density of bus stops (/km^2^)	1.579	1.579	1.581
Density of hospitals (/km^2^)	1.326	1.318	1.330
Average slope (°)	2.664	2.618	2.667
Green space coverage (%)	1.918	1.974	1.783
Density of fast-food outlets (/km^2^)	2.509	2.492	2.526
Density of convenience stores (/km^2^)	4.797	4.783	4.808
Density of supermarkets (/km^2^)	2.547	2.540	2.548
Density of other grocery stores (/km^2^)	4.007	4.000	4.018
Density of other restaurants (/km^2^)	4.359	4.409	4.322
Density of cafés (/km^2^)	3.279	3.175	3.261
Annual average temperature (°C)	2.313	2.224	2.239
Annual total precipitation (mm)	1.883	1.838	1.848

VIF, variance inflation factor; ADI, areal deprivation index.

^a^number of neighborhood areas.

^b^adjusted for the proportions of participants aged 65–74 years and ≥75 years, with participants aged <65 years as the omitted age group, and for the proportion of men.

^c^adjusted for the proportions of participants aged 65–74 years and ≥75 years, with participants aged <65 years as the omitted age group.

## Discussion

This study extends existing research by simultaneously examining multiple neighborhood characteristics associated with hypertension prevalence in Japan, where evidence remains limited. Several socioeconomic and physical environmental characteristics were associated with hypertension prevalence. When accounting for interrelationships among neighborhood characteristics, the proportion of university graduates and green space coverage remained significant, with park density additionally significant among men.

First, socioeconomic characteristics, such as the ADI and the proportion of university graduates, showed significant associations in partial correlation analyses, indicating that neighborhood socioeconomic status may be an important determinant of hypertension among participants. Previous research has shown that not only individual socioeconomic status but also the neighborhood socioeconomic characteristics are associated with the risks of cancer [40], stroke [41], latent atherosclerosis [42], and several other diseases. The present findings demonstrated a similar pattern for hypertension in Japan. These findings suggest that broader neighborhood socioeconomic characteristics may play an important role in hypertension prevalence. Among these factors, the proportion of university graduates in the neighborhood remained significantly negatively associated with hypertension prevalence, even after adjusting for other neighborhood characteristics. This finding can be interpreted in light of previous evidence suggesting that educational attainment reflects individuals’ knowledge, motivation, and abilities rather than serving merely as an indicator of economic resources [43]. Tokuda et al. [44] reported that, among Japanese adults, educational attainment was significantly associated with health literacy, whereas income was not. Therefore, the proportion of university graduates in this study may reflect the collective educational level and health literacy of residents. This represents their capacity to obtain, understand, evaluate, and use information to adopt health-promoting behaviors that prevent lifestyle-related diseases and influence hypertension prevalence.

Furthermore, the correlation analysis revealed significant associations between hypertension prevalence and several physical environmental factors, including average temperature and intersection density. These findings may indicate that the development of the physical environment impacts hypertension prevalence. In particular, green space coverage was significantly associated with hypertension prevalence in both the correlation and regression analyses. Several mechanisms may explain the association between neighborhood green space and hypertension prevalence. Previous systematic reviews have reported that exposure to green spaces is related to increased physical activity, reduced stress, and improved mental health, which may mediate the association between green environments and cardiovascular outcomes [45, 46]. Keith et al. [47] comprehensively reviewed the potential mechanisms linking green spaces and cardiovascular health. In addition to the beneficial effects of green spaces on physical activity and mental health, they summarized several potential pathways, including reductions in air pollution, traffic noise, and light pollution, mitigation of urban heat, exposure to biogenic compounds, enhanced immune function, and improved social interaction and neighborhood social cohesion. Collectively, these multiple pathways may contribute to the lower hypertension prevalence observed in neighborhoods with greater green space coverage in the present study.

In addition, among men, park density was significantly and inversely associated with hypertension prevalence in both the partial correlation and quasi-Poisson regression analyses. Like green spaces, parks provide accessible settings for walking, recreation, and other forms of physical activity, representing one plausible pathway through which neighborhood environments may influence blood pressure [48]. One possible explanation for the male-specific association observed in the present study is sex differences in the frequency and patterns of park use. Previous studies have reported that parks are more frequently used by men than women and that men are more likely to engage in recreational and moderate-to-vigorous physical activity during park visits [49]. Consequently, greater park availability may translate into larger increases in recreational physical activity among men, potentially contributing to the lower hypertension prevalence observed among men in the present study.

Furthermore, in partial correlation analyses adjusted for the proportions of participants aged 65–74 years and ≥75 years, and for the proportion of men in the combined analysis, several neighborhood characteristics, including ADI and bus stop density, were significantly correlated with hypertension prevalence. However, in quasi-Poisson regression analyses that simultaneously included all neighborhood characteristics and accounted for their interrelationships, these associations were no longer significant. These results suggest that associations observed in univariate analyses may have been confounded by correlations with neighborhood characteristics, and their independent effects were attenuated when considered simultaneously. Similarly, a previous study has shown that when multiple neighborhood socioeconomic characteristics are considered simultaneously, the observed associations differ from those identified in individual analyses [23]. The findings of this study further underscore the importance of simultaneously considering multiple neighborhood characteristics when examining their associations with health outcomes.

This study has some limitations. First, it employed a community-level correlational design. Previous research has demonstrated that associations observed at the community level do not necessarily correspond to those at the individual level [50]. Future research should therefore incorporate multilevel analytical approaches to simultaneously assess associations at both the individual and neighborhood levels. Second, the study was cross-sectional. Although the proportions of participants aged 65–74 years and ≥75 years were statistically controlled, the possibility of reverse causation cannot be fully ruled out, as individuals with certain baseline characteristics, such as hypertension, may have been disproportionately concentrated in particular areas. Longitudinal or cohort studies are needed to establish causal relationships. Third, hypertension prevalence was calculated based on diagnostic codes rather than directly measured blood pressure or medication information. Therefore, individuals with undiagnosed or untreated hypertension may not have been captured, potentially leading to an underestimation of hypertension prevalence and misclassification of the outcome. Future studies using clinically measured blood pressure and medication information are needed to validate and extend our findings. Fourth, potential selection bias may exist. The geographic distribution of participating municipalities was uneven, with greater representation from western regions, which may limit the generalizability of the findings. Nevertheless, these findings contribute to a deeper understanding of regional health disparities in Japan and other developed Asian contexts. From an urban planning and public health perspective, they also provide valuable insights into regions and policy domains that should be prioritized in health promotion efforts.

## Conclusions

This study showed that socioeconomic and physical environmental factors at the neighborhood level were associated with hypertension prevalence in Japan. Neighborhood educational attainment and green space coverage appeared to play important roles in shaping regional differences in hypertension prevalence. Future studies should employ multilevel analysis based on longitudinal data from randomly selected populations.

## Abbreviations

GIS: geographic information system
LIFE Study: Longevity Improvement & Fair Evidence Study
ADI: areal deprivation index
NHI: National Health Insurance
MCSE: Medical Care System for the Elderly
NDVI: Normalized Difference Vegetation Index
SD: standard deviation

## Declarations

### Availability of data and materials

Data cannot be shared for privacy or ethical reasons.
